# Evaluation of metabolic uptake in gynecological organs using FDG-PET in women diagnosed with nongynecological malignancies

**DOI:** 10.55730/1300-0144.6037

**Published:** 2025-02-14

**Authors:** Funda ATALAY, Uğurcan ZORLU, Koray ASLAN, Fatma BATAK, Tuba ZENGİN AKSEL, Gülin UÇMAK

**Affiliations:** 1 Department of Gynecologic Oncology, University of Health Sciences, Dr Abdurrahman Yurtaslan Ankara Oncology Training and Research Hospital, Ankara, Turkiye; 2 Department of Gynecologic Oncology, Etlik City Hospital, Ankara, Turkiye; 3 Department of Nuclear Medicine, University of Health Sciences, Dr Abdurrahman Yurtaslan Ankara Oncology Training and Research Hospital, Ankara, Turkiye

**Keywords:** FDG-PET, gynecological malignancies, SUVmax, metabolic imaging, endometrial thickness

## Abstract

**Background/aim:**

Gynecological malignancies, including those affecting the uterus, cervix, vagina, vulva, and adnexa, pose significant physical and psychosocial burdens. Early detection and effective management of these malignancies are critical for improving outcomes. This study aims to evaluate metabolic uptake patterns in gynecological organs using fluorodeoxyglucose positron emission tomography (FDG-PET) and to analyze their malignancy potential in women with nongynecological cancers.

**Materials and methods:**

A retrospective analysis was conducted on 221 women with nongynecological malignancies who exhibited pathological FDG uptake in gynecological organs on FDG-PET/CT imaging. Lesions were evaluated based on the standardized uptake value maximum (SUVmax), morphological characteristics on contrast-enhanced CT, and further gynecological assessment using ultrasonography, biopsy, and endometrial sampling. Statistical analyses, including the receiver operating characteristics curve and descriptive statistics, were performed using SPSS software, with significance set at p < 0.05.

**Results:**

Pathological FDG uptake was observed in the uterus (60.6%), adnexa (30.3%), cervix (14.02%), vulva (4%), and vagina (2.2%). The mean SUVmax of lesions varied across sites, with uterine lesions showing a mean SUVmax of 6.96 ± 3.55. An SUVmax cutoff of >10.11 predicted malignancy in uterine lesions with 86% sensitivity and 82% specificity. Among patients with uterine involvement, malignancy was confirmed in 10 cases, all of whom were on tamoxifen therapy. Endometrial thickness was significantly higher in malignancy cases (10.6 mm vs. 5.8 mm, p = 0.014). Ultrasonography and biopsy findings largely confirmed the benign nature of other lesions, highlighting the role of multimodal diagnostic approaches.

**Conclusion:**

FDG-PET imaging is a valuable tool for identifying metabolic activity in gynecological organs and for differentiating malignant lesions from benign ones. High SUVmax values and endometrial thickness are significant indicators of malignancy, particularly in patients undergoing hormonal therapy. This study underscores the importance of integrating metabolic imaging with clinical and morphological assessments for the early detection and management of gynecological malignancies.

## 1. Introduction

While women’s health occupies a significant place in general medical research, malignancies observed in women constitute a distinct area of interest. Malignancies of the uterus, cervix, vagina, vulva, and adnexa not only affect physical health but also lead to psychosocial impacts, significantly reducing individuals’ quality of life [1]. Gynecological cancers represent an ongoing source of concern, due to their persistently high incidence and cancer-related mortality. Specific protocols are applied in order to decrease incidence and progression of these cancers. [2]

Fluorodeoxyglucose positron emission tomography (FDG-PET) has been increasingly utilized in oncology in recent years as an imaging modality. FDG-PET plays a critical role, particularly in the diagnosis, staging, and evaluation of treatment response in malignancies. The uptake of FDG by cells with high metabolic activity provides essential information for assessing tumor spread and progression in oncology patients [3]. For obstetricians and gynecologists, interpreting FDG-PET results in patients showing signs of malignancy represents a key step in the diagnosis and follow-up of gynecological cancers.

In the gynecologic oncology literature, the presence of metabolic uptake in the abdominal and pelvic regions, as assessed via FDG-PET, offers valuable insights into the prognosis of malignancies in these areas [4]. Furthermore, comparing the uptake detected by FDG-PET with diagnostic methods such as ultrasonography, HPV testing with smear, endometrial sampling, colposcopy, endocervical curettage (ECC), and hysteroscopy has the potential to provide a more personalized approach in clinical management.

In this study, metabolic uptake in the uterus, cervix, vagina, vulva, and adnexa, detected after FDG-PET imaging in women diagnosed with malignancies, was systematically evaluated. Subsequently, a detailed analysis of the presence and malignancy potential of these uptakes was conducted. The findings are expected to contribute to the existing body of knowledge in the field of gynecologic oncology and guide clinical practices.

## 2. Materials and methods

This study was conducted on patients with a clinically confirmed history of primary nongynecological malignancies who were followed at Ankara Oncology Hospital. The study included individuals who showed metabolic uptake in gynecological organs (uterus, cervix, vagina, vulva, and adnexa) on FDG-PET imaging results and were referred to the gynecological oncology clinic for further consultation based on these findings.

All FDG-PET/CT images underwent evaluation by specialists in nuclear medicine. Initially, a review was conducted of low-dose, noncontrast CT images and FDG-PET images. The areas of maximum FDG uptake in the gynecological lesions were delineated using a circular region of interest (ROI), and SUVmax values were quantified. The SUV was determined using the formula: [decay-corrected activity (MBq) / tissue volume (mL)] / [injected FDG dose (MBq) / body mass (g)].

Full-dose contrast-enhanced CT images were subsequently examined to evaluate the size and morphological characteristics of all gynecological lesions. The gynecological lesions were categorized into cystic-dominant and solid-dominant types. The cystic-dominant type is characterized by a cystic component comprising more than fifty percent of the lesion, while the solid-dominant type is defined by a solid component exceeding fifty percent of the lesion. The enhancement of the cystic wall or solid components of the gynecological lesion was analyzed through comparison with contrast-enhanced CT images. Lesions characterized by cystic dominance, exhibiting notable enhancement, septations, or solid components on contrast-enhanced CT images, were categorized as malignant gynecological lesions.

Patients underwent gynecological evaluation procedures, following standard clinical protocols to ensure detailed assessment. In the first stage, gynecological ultrasonography was performed to determine the size and characteristics of existing lesions. Ultrasonography findings were evaluated alongside the patients’ general clinical condition, and in necessary cases, HPV testing with smear was conducted. This test was utilized as an important screening tool to identify cervical pathology. Based on smear results, additional invasive procedures such as colposcopy and ECC were performed in cases of abnormal cervical findings.

Endometrial biopsy was carried out to assess conditions such as endometrial cancer and hyperplasia, with hysteroscopy being used to support the procedure when necessary. This multistep evaluation process provided a comprehensive and systematic approach to determining the malignancy potential of metabolic uptake in the patients’ gynecological organs.

The research covered patients who presented at Ankara Oncology Hospital between 2020 and 2024 and were referred for evaluation based on their FDG-PET results. The findings aim to contribute to the improvement of current practices in gynecologic oncology evaluation and management.

Statistical analysis was performed using IBM SPSS Statistics, v. 23.0 (IBM Corp., Armonk, NY, USA). The analysis included descriptive and inferential statistics. Mean (χ̄) and standard deviation (SD) were used to analyze continuous data. Frequency (n) and percentage (%) values were used to describe categorical variables. Differences in variable characteristics between groups were assessed using Student’s t-test for continuous variables and the chi-square (χ^2^) test for categorical variables.

A receiver operating characteristic (ROC) curve was generated to determine the optimal cut-off point for differentiating premalignant and malignant lesions from benign lesions. The area under the curve (AUC) was calculated for SUVmax and for the combined parameters of SUVmax and cyst wall thickness. The findings included hazard ratios and 95% confidence intervals (CI). The analysis employed a statistical significance level of p < 0.05.

## 3. Results

Our study was conducted with a total of 221 patients. Among these, 178 patients had primary breast cancer, 10 had colon cancer, nine had lymphoma, six had lung cancer, four had thyroid cancer, three had bladder cancer, two had neuroendocrine tumors, two had multiple myeloma, and one patient each had osteosarcoma, plasmacytoma, nasopharyngeal cancer, pancreatic cancer, synovial sarcoma, tongue cancer, and Ewing’s sarcoma.

The youngest patient was 24 years old, and the oldest was 84 years old, with a mean age of 48.8 ± 16.9 years.

Of the patients, 36.65% (81 patients) received chemotherapy. Surgical treatment was performed on 53.39% of the patients (118 patients), and 23.07% (51 patients) received radiotherapy. Among the patients followed for breast cancer, 39 patients (21.91%) were receiving tamoxifen therapy.

When the PET/CT scans were examined, pathological FDG uptake was detected in the uterus in 134 patients (60.6%). Pathological FDG uptake was observed in the cervical region in 31 patients (14.02%), and in the adnexal region in 67 patients (30.3%). Pathological FDG uptake was detected in the vulva in nine patients and in the vagina in five patients (4% and 2.2%, respectively).

Regarding the PET/CT findings, the mean SUVmax across all lesions was 7.14 ± 2.91, with the highest value being 31.9 and the lowest 2.4. The mean SUVmax of uterine lesions was 6.96 ± 3.55, with a maximum of 31.9 and a minimum of 3.2. The mean SUVmax for adnexal uptake was 6.35 ± 3.01, with a maximum of 15.5 and a minimum of 2.4. Cervical uptake had a mean SUVmax of 8.2 ± 3.14, with a maximum of 17.9 and a minimum of 3.4. Vaginal uptake had a mean SUVmax of 8.105 ± 3.9, with a maximum of 17.9 and a minimum of 2.7. Vulvar uptake had a mean SUVmax of 6.95 ± 2.02, with a maximum of 8.8 and a minimum of 5.9 (Table 1). Some patients exhibited multiple organ involvement, and in cases with more than one FDG uptake within the same anatomical region, the highest SUVmax value was taken into consideration.

Among patients with uterine involvement, pathological examination of endometrial samples from 10 individuals revealed findings suggestive of malignancy (one case of breast cancer metastasis, one case of endometrial intraepithelial neoplasia, and eight cases of endometrial carcinoma). The mean SUVmax for these patients was 11.93 ± 4.09, with a maximum of 31.9 and a minimum of 4.7. In patients with uterine involvement but without malignancy detected through endometrial sampling, the mean SUVmax was 6.55 ± 3.43. The difference in SUVmax values between these two groups was statistically significant (p = 0.021). All patients in whom malignancy was detected through endometrial sampling were receiving tamoxifen therapy. According to the ROC analysis, a SUVmax value greater than 10.11 predicts malignancy with a specificity of 82% and a sensitivity of 86% in cases of uterine involvement.

When considering endometrial thickness, the mean value was 10.6 mm in the malignancy group and 5.8 mm in the no malignancy group. This difference was also statistically significant (p = 0.014).

Upon sonographic evaluation, 21 patients with uterine involvement were found to have uterine fibroids (myoma uteri). The mean SUVmax was 7.04 ± 3.44 in patients with myoma uteri and 6.94 ± 3.59 in those without. The difference between the two groups was statistically significant (p = 0.039).

The mean SUVmax in patients with endometrial polyps detected through pathological examination was 7.12 ± 3.98, compared to 6.88 ± 3.41 in those without polyps. The difference between the groups was not statistically significant (p = 0.124) (Table 2).

In the sonographic examination, ovarian cysts were detected in 44 patients (65.67%) with ovarian involvement, all of whom exhibited benign characteristics. No pathological findings were observed.

Additionally, no malignancy-related findings were detected in the results of the smear, ECC, or colposcopy. Similarly, no evidence of malignancy was found in vulvar and vaginal biopsies.

## 4. Discussion

This study highlights the critical role of FDG-PET imaging in assessing metabolic activity in gynecological organs of women diagnosed with nongynecological malignancies. Previous investigations have underscored the increasing utility of FDG-PET in detecting early-stage malignancies, particularly due to its ability to visualize regions of high metabolic activity [3]. Our findings demonstrate that pathological FDG uptake is common, with 60.6% of patients exhibiting abnormal metabolic activity in the uterus, which is suggestive of potential malignancies requiring further diagnostic exploration.

### 4.1. Metabolic uptake and malignancy detection

The mean SUVmax observed in our study was 7.14 ± 2.91, aligning with existing literature that emphasizes the correlation between elevated SUVmax values and malignancy risk. Notably, patients presenting with an SUVmax greater than 10.11 showed 86% sensitivity and 82% specificity for detecting malignancy, suggesting this as a potential threshold for clinical decision-making in the evaluation of endometrial lesions. Previous studies have also established critical SUVmax cut-off values that aid in differentiating malignant from benign lesions, reinforcing the need for standardized protocols in gynecological oncology [5,6].

Moreover, the detailed pathological assessment of endometrial samples from 10 patients with confirmed malignancy adds weight to our findings. Among these, the presence of eight cases of endometrial carcinoma signifies a considerable risk in this patient population, particularly among those undergoing hormonal therapy such as tamoxifen. Tamoxifen, primarily used in the treatment of breast cancer, has been implicated in the development of endometrial hyperplasia and carcinoma [7]. By identifying nine patients on tamoxifen therapy who were diagnosed with malignancies, this study highlights the pressing need for vigilant monitoring in women receiving hormonal therapy, as they may have a heightened risk of developing secondary malignancies in the endometrial region.

### 4.2. Endometrial thickness as a prognostic indicator

Additionally, our analysis revealed a statistically significant difference in endometrial thickness between the malignancy and no malignancy groups (10.6 mm vs. 5.8 mm, p = 0.014). This finding is consistent with established guidelines, which suggest that abnormal endometrial thickness is a reliable indicator for further evaluation in postmenopausal women, as it correlates with malignancy risk. The clinical value of measuring endometrial thickness should be emphasized, particularly in populations at risk due to previous malignancies or hormonal therapy.

### 4.3. Sonographic evaluations and lesion characteristics

In conjunction with PET/CT findings, ultrasound evaluations revealed benign characteristics in most of the detected ovarian cysts (65.67%), with no malignancy reported in pathology results from cervical smears or biopsies of the vulva and vagina. This discrepancy highlights the importance of multimodal approaches in diagnostic evaluation. While FDG-PET may identify areas of metabolic activity requiring further investigation, imaging modalities such as ultrasound provide essential context for evaluating lesion characteristics [4]. Hence, future studies should explore more structured protocols that integrate FDG-PET findings with ultrasound results to enhance diagnostic accuracy.

Our classification of lesions into cystic-dominant and solid-dominant types further enhances the diagnostic framework. Solid-dominant lesions with increased uptake on CT imaging were classified as malignant, a classification supported by previous studies linking solid components to adverse outcomes [8,9]. This stratification enables tailored management strategies for patients with varying lesion characteristics.

### 4.4. Limitations and future directions

However, this study has limitations. The single-center design may limit the generalizability of the findings. Additionally, a larger-scale study involving a more diverse cohort would strengthen our understanding of the relationship between metabolic activity and gynecological malignancies. Furthermore, the retrospective nature of certain analyses may introduce selection bias, potentially affecting the interpretation of outcomes.

Future research should aim to evaluate the longitudinal outcomes of patients based on SUVmax measurements and to integrate advanced imaging modalities such as MRI or newer PET tracers that may further distinguish benign from malignant processes. Understanding the molecular pathways influencing FDG uptake could also help elucidate the mechanisms underlying false-positive findings in benign conditions, thereby advancing diagnostic capabilities [10].

Our findings demonstrate that FDG-PET is a valuable tool for assessing gynecological lesions in women with nongynecological malignancies. Integrating metabolic imaging data with clinical and morphological analyses provides a comprehensive approach that may enhance early detection rates and guide treatment planning. As evidence-based protocols continue to evolve, the role of FDG-PET in management of gynecological malignancies is likely to become increasingly significant.

## 5. Conclusion

This study evaluated the metabolic utility of FDG-PET imaging in assessing gynecological organs in women diagnosed with nongynecological malignancies. The findings demonstrate that FDG-PET plays a significant role in the diagnosis and management of gynecological lesions. Notably, pathological FDG uptake in the uterine region was observed in 60.6% of cases, clearly indicating the value of FDG-PET in the early detection of gynecological malignancies.

Moreover, SUVmax values greater than 10.11 were strongly associated with malignancy, suggesting that this threshold could serve as a clinical cutoff point for practitioners. The results of endometrial biopsies indicated that tamoxifen use may increase the risk of endometrial malignancy, reinforcing the importance of regular monitoring and early screening strategies in this patient group.

Endometrial thickness measurements demonstrated a significant association with malignancy, supporting the view that endometrial evaluation is a critical component of care in postmenopausal women. However, discrepancies between ultrasonography and FDG-PET findings underscore the importance of multimodal diagnostic approaches in clinical practice.

In conclusion, FDG-PET evaluations provide valuable insights for physicians in the diagnosis and management of gynecological malignancies, contributing to the growing body of knowledge in gynecologic oncology. This study aims to further clarify the role of FDG-PET in managing gynecological cancers, with future validation expected from larger-scale research.

## Figures and Tables

**Table 1:** Mean SUVmax values by site of involvement.

Site of involvement	Mean SUVmax	Minimum SUVmax	Maximum SUVmax
Uterus	6.96 ± 3.55	3.2	31.9
Adnexal region	6.35 ± 3.01	2.4	15.5
Cervical region	8.2 ± 3.14	3.4	17.9
Vagina	8.105 ± 3.9	2.7	17.9
Vulva	6.95 ± 2.02	5.9	8.8

**Table 2:** Mean SUVmax values in uterine involvement.

Condition	Mean SUVmax	p-value
Malignancy present	11.93 ± 4.09	0.021
Malignancy absent	6.55 ± 3.43	
Myoma present	7.04 ± 3.44	0.039
Myoma absent	6.94 ± 3.59	
Polyp present	7.12 ± 3.98	0.124
Polyp absent	6.88 ± 3.41	
